# Cross-country variations in the caregiver role: evidence from the ENTWINE-iCohort study

**DOI:** 10.1186/s12889-024-18302-6

**Published:** 2024-03-26

**Authors:** Mikołaj Zarzycki, Noa Vilchinsky, Eva Bei, Giulia Ferraris, Diane Seddon, Val Morrison

**Affiliations:** 1 Department of Psychology, Liverpool Hope University, Liverpool, United Kingdom; 2 Department of Psychology, Faculty of Social Sciences, Bar-Ilan University, Ramat Gan, Israel; 3 Department of Political and Social Sciences, University of Bologna, Bologna, Italy; 4 Department of Health Psychology, University Medical Center Groningen, University of Groningen, Groningen, The Netherlands; 5 School of Medical and Health Sciences, Bangor University, Bangor, Wales United Kingdom; 6 School of Psychology and Sports Sciences, Bangor University, Bangor, Wales United Kingdom

**Keywords:** Informal caregiving, Cross-country analysis, Culture, Society, Caregiver values, Meaning in life, Illness beliefs, Motivations and willingness to care, Caregiver outcomes

## Abstract

**Background:**

Globally, economically developed countries face similar ageing demographics and the challenge of a ‘care gap’, yet they vary due to different care and formal support systems, and different cultural and societal norms around illness and care. The aim of this exploratory study was to examine cross-country variations in caregiver motivations, willingness, values, meaning in life, illness beliefs, and experiences of wellbeing, gain, health-related quality of life, burden and depression, across 6 European countries and Israel. Cross-country differences in the above-mentioned informal caregiver experiences are rarely described.

**Methods:**

An online survey (ENTWINE-iCohort) was conducted using validated measures wherever possible. This paper utilises data from 879 caregivers and seven countries (Greece, Italy, the Netherlands, Poland, Sweden, the UK, and Israel).

**Results:**

No consistent finding supporting the concurrent relationship between caregiver support policies/country culture and caregiver motivations/willingness was found. Caregivers in countries typically characterised by individualist cultures reported lower familism, higher self-enhancement values, and greater perceived illness threat compared to more collectivist countries. Search for meaning was higher in poorer countries than in wealthier countries. Higher negative caregiver experiences (e.g., burden) and lower positive experiences (e.g., wellbeing) were generally observed in countries with underdeveloped caregiver support as compared to countries with more developed formal support systems.

**Conclusions:**

Cross-country variations can be explained to varying degrees by national policies around care (or their absence) and country cultural contexts. The results emphasise the importance of formal support services for achieving positive caregiver experiences, and help inform the development of policies and measures to support caregivers in Europe and Israel.

## Background

Informal caregivers provide care for family members or friends who have care and support needs consequent to ageing, illness or health challenge(s) [1]. In Europe, an estimated 20–44% of individuals perform informal care [2], forming the backbone of health and social care delivery worldwide [3]. However, demographic changes and the ‘care gap’ pose risks to the sustainability of informal caregiving. The caregiver-care recipient support ratio is projected to decline in nearly all European countries, from 6 potential caregivers per care recipient in 2011 to 2 potential caregivers by 2050 [4].

The health and social care policies underpinning family support systems and the cultural values influencing the provision of informal care vary widely between countries [5]. Aranda & Knight [6] found cultural differences in caregiver appraisals, coping and use of social support which affected caregiver outcomes [7, 8], however it is only in more recent years that cross-country analyses of informal caregiving have been documented [5, 9–11]. Cross-country differences remain rarely described when it comes to caregiver motivations, willingness, values, meaning in life, illness beliefs, and caregiver experiences of wellbeing, gain, health-related quality of life, burden and depression. It is necessary to consider the contextual factors that may influence caregiver experience, in particular the different care systems and family culture that may exist across different countries [10, 12–14] as these may inform caregiver experiences. The current exploratory paper endeavours to investigate variations in caregiving experiences across different countries, focusing on caregiver motivations, and willingness to care, values, meaning in life, illness beliefs, wellbeing, gain, health-related quality of life, burden, and depression. The cross-country variations in the above-mentioned psychosocial variables comprise a major lacuna in the caregiving literature and should be documented.

The constructs of *motivations* and *willingness to provide care* are essential to our understanding of the caregiving experience [15]. Caregiving motivations are typically conceptualised as either extrinsic or intrinsic, especially in the caregiving literature [15–17]. Intrinsic motivations emerge from internal influences (e.g., emotional bonding), extrinsic motivations from external influences (e.g., social expectations). *Willingness to provide care* refers to a caregiver’s attitude towards providing various forms of support for an individual, whether this relates to a current or future need [18]. We have limited understanding of the extent to which motivations and willingness to provide informal care vary between countries.

Prior research has identified differences and similarities in cultural and personal values across countries [e.g., 19, 20], including familism [7]. Examined in the context of caregiving, *familism* is a multidimensional construct incorporating values of familial piety, felt responsibility and familial obligation [7]. Different countries have differing family cultures which underpin the value of familism [10, 12, 13]. *Personal values* are broad beliefs that serve as guiding principles in a person’s life with ‘basic’ universally-valid values including those of power, achievement, benevolence, or universalism [21]. These basic values can form ‘higher-order’ values, such as self-enhancement values (comprising power and achievement values) which oppose self-transcendence values (comprising universalism and benevolence values), reflecting self-interest versus concern for others’ welfare [21].

*Meaning in life* is one of the oldest constructs examined by psychologists [22, 23]. Although cross-country variations in the meaning in life have been investigated [24], the construct has not been examined in the context of caregiving. A global meaning (meaning in life), rather than a situational meaning (meaning in caregiving only), was investigated in two distinct and independent domains: the *presence of meaning* which captures the subjective sense that one’s life is meaningful, and the *search for meaning* which measures the drive and orientation toward finding meaning in one’s life [25].

*Beliefs about health and illnesses* are culturally bound [14]. For instance, a person with cultural beliefs rooted in karma may see caregiving as repaying debts from previous lives [26]. Qualitative meta-synthesis [14, 27] has evidenced illness perceptions to be determinants of caregiver experience (i.e., motivations and willingness to provide care) and potential cross-cultural differences in illness perceptions. Cultural, societal or illness epidemiology differences between countries may lead to different caregiver illness perceptions, including the perception of the care recipient’s *illness threat* [28]. Literature on between-country variations in perceived illness threat is limited amongst patient groups [e.g., 29], and in terms of variations in caregivers’ perceptions of their care recipients’ health condition(s). Illness perceptions, including perceived illness threat, may have an important impact on caregivers’ and care recipients’ wellbeing [30, 31].

Caregivers can experience both positive and negative experiences as a result of their caregiving [32, 33]. These experiences may differ by national contexts since care is differently organised across different countries. Informal care provision is also a dynamic and multifaceted experience that occurs in a sociocultural context, and changes over time in response to many factors. Factors such as gender and age have been linked to variation in caregiving experiences [e.g., 34–36], but little is known about between-country variations [5, 12, 37], including variations in wellbeing, gains, health-related quality of life, burden, and depression. A European multinational cohort study conducted in 8 countries reported multiple cross-country differences in caregiver burden and health-related quality of life, and related these differences to national health and social care systems [5]^1^. Another multinational study conducted in 8 European countries documented variations in caregiver burden and quality of life across countries although psychological wellbeing was found to be similar [9]. In this study the biggest differences were seen between southern European countries (e.g., Spain) and northern European countries (e.g., Sweden) with southern countries reporting higher caregiver burden than northern countries. These differences, as these authors propose and as we hypothesise below, may be related to differences in health and social care systems [9].

### Country context underpinning caregiving

This paper draws on data from the ENTWINE-iCohort - a multinational study designed to explore caregivers’ experiences in the context of chronic health conditions [38]. Caregiving is embedded in a specific national context, shaped by different historical, political and economic circumstances and inevitably countries vary in the extent to which informal caregivers are supported by public policies [39, 40]. Countries in the current sample (ENTWINE-iCohort)^2^ included Greece, Israel, Italy, the Netherlands, Poland, Sweden, and the UK, and these countries have different forms of caregiver support in place.

Anderson [13] proposes a classification of caregiver support systems, distinguishing between countries with an informal care-led model (family-based model), and countries with a service-led model. This dichotomous classification has been applied within previous cross-country studies on informal care where family-based countries were defined as those with a strong role of family as the main supplier of care whilst service-based countries were those where the state provides most of the care, with statutory support widely offered [10, 12]. Other classifications, such as those provided in national policies for adult caregivers across Europe and published by the European Commission [39, 40] distinguish more categories of country based upon the support available to informal caregivers, ranging from countries characteristic of *developed caregiver support* to countries with *underdeveloped caregiver support* [39, 40]. Table 1 below presents a short classification of countries included in the ENTWINE-iCohort based on these two categorisations of caregiver support systems [13, 39, 40]. In general, provision of care services is found to be lower and informal care higher in more southern and eastern European countries [5, 41, 42].

### The cultural context across countries: individualist-collectivist cultures of countries

When exploring national variations in caregiving, it is important to acknowledge the potential contribution of the dimensions of collectivism-individualism, described particularly within cultural psychology [43]. Individualist cultures are characterised by a focus on individual needs and relative detachment from relationships and communities whereas collectivist cultures are defined by the importance of relationships, roles and status within a social system. Based on a comparison across 65 countries, scaled (scale range 0-100) on a collectivism–individualism continuum and where higher scores indicate more individualist cultures, lower scores indicate more collectivist cultures, Hofstede and colleagues [44] found the following scale scores: the United Kingdom (89), the Netherlands (80), Italy (76), Sweden (71) scored high on individualism, whereas Poland (60) and Israel (54) received medium scores, with Greece scoring the lowest (35).

Table 1 below presents a short classification of countries analysed in this article and included in the ENTWINE-iCohort based on the above-mentioned categorisations of caregiver support systems [13, 39, 40] and cultural dimensions of collectivism-individualism [44].

**Table 1:** Policy and cultural country characterisation (ENTWINE-iCohort)

Country	Care specific characterisation	Collectivism-individualism score (scale range: 0-100)
Greece	Family-based	35
Israel	Mixed	54
Italy	Mixed	76
Netherlands	Mixed	80
Poland	Family-based	60
Sweden	Service-based	71
United Kingdom	Mixed	89

Note: Higher scores for collectivism-individualism indicate more individualist cultures

### Study aims and hypothesis

The aim of the current study was to examine cross-country variations for six European countries and Israel, focusing on the following variables: caregiver motivations, willingness, values, meaning in life, perceived illness threat, and caregiver experiences of wellbeing, gain, health-related quality of life, burden and depression. Based on the above reviewed limited evidence base, we hypothesised that there would be greater negative impact of caregiving in family-based, informal care-led countries compared to in service-based countries due to higher caregiving responsibilities/reduced receipt of formal support.

## Methods

### Participants and procedures

This study draws data from a larger study, the ENTWINE-iCohort, and employed an online, convenience cross-sectional sample of adult caregivers from 8 European countries and Israel [38]. Low-recruiting countries, Germany and Ireland (*N* = 25, *N* = 42, respectively), were excluded from the analyses presented in this article as they did not meet the necessary assumptions. To be eligible caregivers had to be 18 years or over and be providing care for a family member or a friend with a chronic health condition, disability, or any other care need. Data collection took place from August 2020 until August 2021, with varying start points across countries [38]. The response rate for the survey, defined as the number of fully completed surveys by the number of invitation emails sent, was 42%. Full primary ethical approval was obtained from the School of Psychology Academic Ethics Committee at Bangor University, Wales, UK, for non-clinical recruitment, and the NHS Research Ethics and Governance Committee for clinical site recruitment (20/WA/0006). English language documents were translated, and approvals sought and received in all participating countries as required by national legislations.

### Measures

The list of all measures included in the ENTWINE-iCohort survey is reported in the protocol paper [38]. Those used in the current analysis are described below.

Informal caregivers’ background variables included *socio-demographic variables* such as: age, gender, country of residence, partnership status, level of education, relationship t*y*pe of the caregiver to the care recipient, employment status, religious affiliation, ethnicity, income, cash benefits, care recipient’s age, gender, type, and duration of health conditions (care recipient variables measured based on caregiver reports). The *caregiving context* addressed: the length of the caregiving, caregiver health status, care recipient’s health condition, previous caregiver experience, the presence of other caregivers/paid care workers, living arrangements, intensity of care. The ability of the care recipients to perform Activities of Daily Living (ADL) independently (i.e., bathing, dressing, toileting, transferring, feeding) was assessed by caregivers by filling in the *Katz Index* [45]. The scale consists of 6 items, and participants score either yes (1) or no (0) for independence in each ADL, therefore a maximum score of 6 indicates full function/independence, 4 indicates moderate functional impairment, and 2 or less indicates severe functional impairment. This measure demonstrated good internal consistency (Cronbach’s α = 0.86).

*Motivations to provide care.* Caregivers’ motivations to provide care were measured by the Motivations in Elder Care Scale [16], which is comprised of two subscales: Extrinsic Motivations to Care (EXMECS) and Intrinsic Motivations to Care (INMECS). Designed for caregivers of older adults, the wording of the questions was amended to be appropriate for the general population of adult caregivers. The Motivations in Elder Care Scale used to assess caregiver motivations was created by British scholars [16], and was translated for the first time to other languages for the current study. The EXMECS subscale measured extrinsic motivations and consists of seven items, each rated on a five-point scale ranging from ‘Strongly disagree’ (1) to ‘Strongly agree’ (5), with a higher score (maximum score = 35) indicating greater extrinsic motivations to provide care. The INMECS subscale measured intrinsic reasons for providing care and consists of six questions, each rated on a five-point scale ranging from ‘Strongly disagree’ (1) to ‘Strongly agree’ (5), with a higher score (maximum score = 30) indicating greater intrinsic motivations to provide care. Both the EXMECS and the INMECS demonstrated good internal consistency (Cronbach’s α = 0.77; Cronbach’s α = 0.80 respectively).

*Willingness to provide care.* The Willingness to Care Scale [18] was used to assess caregiver’s willingness to provide care and consists of three subscales of 10 *emotional* tasks (e.g., comfort when the care recipient is sad), 10 *instrumental* tasks (e.g., do the care recipient’s laundry) and 10 *nursing* tasks (e.g., turn the care recipient in bed) typically carried out by caregivers. Caregivers rate each item on a five-point Likert scale from ‘completely unwilling to complete the task’ (1) to ‘completely willing’ (5), with scores computed by the mean of the responses associated with appropriate global scale or subscale (maximum score = 5). The internal consistency was very good for nursing willingness (Cronbach’s α = 0.93) and emotional willingness (Cronbach’s α = 0.92), and good for instrumental willingness (Cronbach’s α = 0.88).

*Familism.* The 21-item Revised Familism Scale (RFS) [46] measured caregivers’ sense of familism and consists of three subscales: familial interconnectedness (12 items, maximum score of 48), familial obligations (5 items, maximum score of 20), extended family support (4 items, max score of 16). Responses were scored on five-point Likert scales from ‘very much in disagreement’ (0) to ‘very much in agreement’ (4). Items were summed for each subscale and all subscales summed to achieve the overall familism score (maximum score = 84 indicating a higher level of familism). The internal consistency was good for familial interconnectedness (Cronbach’s α = 0.77) and extended family support (Cronbach’s α = 0.71) scales and moderate for the familial obligations subscale (Cronbach’s α = 0.62).

*Personal values.* Personal values were assessed using the Portrait Values Questionnaire (PVQ-21) [47], specifically two ‘higher-order values’ subscales: self-enhancement (4 items) and self-transcendence (5 items) derived from Schwartz’s theory of values [48]. Responses were scored on six-point Likert response scales from ‘very much like me’ (1) to ‘not like me at all’ (6). Self-enhancement values were measured by the mean of 2 items pertaining to values of power (maximum score of 6) and 2 items pertaining to achievement (maximum score of 6). Self-transcendence values are measured by the mean of 2 items pertaining to values of benevolence (2 items, maximum score of 6) and 3 items pertaining to universalism (maximum score of 6). The internal consistency was good for self-enhancement values (Cronbach’s α =.74) and moderate for self-transcendence values (Cronbach’s α =.68).

*Meaning in life.* The Meaning in Life Questionnaire (MLQ) [25] consists of 10 items measuring presence of, and the search for, meaning in life. Participants rated items on a seven-point Likert scale from ‘Absolutely untrue’ (1) to ‘Absolutely true’ (7). Higher scores indicate greater meaning in life. The MLQ yields two scores, presence of meaning (5 items, maximum score of 35) and search for meaning (5 items, maximum score of 35). Both subscales demonstrated good internal consistency (Cronbach’s α = 0.85, α = 0.88, respectively).

*Illness threat.* The Brief Illness Perception Questionnaire (B-IPQ) [49] was used to assess caregiver’s perception of the care recipient’s illness threat. The B-IPQ consists of nine items, each assessing one dimension of illness perceptions (e.g., illness consequences, illness timeline, illness concern). The last item is a categorical casual item and does not comprise a total score. The other item responses were scored on a scale from 1 to 10 (modified response range). The total score was generated by summing up the scores for the B‐IPQ items with a reverse scoring of items 3, 4 and 7. A higher total score (maximum = 80) reflects a more threatening perception of illness. The B-IPQ is designed for use across illness populations, with the option to adapt question wording to the specific illness condition [49]. A slight modification was made to the B-IPQ to fit the context relevant to caregivers, with items reworded to indicate to the caregivers to answer the questions with respect to the care recipient’s illness/health condition. Given that there is only one item for each dimension of illness perception (and less than 10 items overall), inter-item correlations were computed to evaluate scale reliability. The mean inter-item correlation amounted to 0.12 which is acceptable and close to the ranges of 0.15-0.50 [50] which indicate the optimal level of scale consistency (when the number of items is less than 10).

*Wellbeing.* Caregiver wellbeing was measured using the five-item World Health Organisation-Five Wellbeing Index (WHO-5) [51]. Items are rated on a six-point scale from ‘at no time’ (0) to ‘all of the time’ (5), which are then summed (raw score ranges from 0 to 25) and transformed into a percentage score (to obtain a percentage score ranging from 0 to 100, the raw score is multiplied by 4). Higher scores indicate better wellbeing (with the score of 100 representing best possible wellbeing). For the current sample, the internal consistency was very good (Cronbach’s α = 0.90).

*Gains.* Caregiver gains were measured using the 10 item GAINS Scale [52]. The 10 items are measured on a four-point Likert scale, 0–3 (maximum score = 30). Higher scores indicate greater gains. In the current study the measure demonstrated good consistency (Cronbach’s α = 0.86).

*Health-related quality of life.* The EQ-5D-5 L [53] was used to assess caregiver health-related quality of life. The EQ-5D is a generic and standardized health-related quality of life instrument, applicable to a wide range of conditions and treatments. Five dimensions of health state are assessed using 5 items: mobility, self-care, usual activities, pain/discomfort, and anxiety/depression. Each dimension has 5 levels: no problems, slight problems, moderate problems, severe problems, and extreme problems. The digits for the 5 dimensions can be combined in a 5-digit code describing the patient’s health state. A total of 3125 combinations with different health states are possible. These may be converted into a country-specific single index value using country specific value sets, which have been derived from large country-specific validation studies using time-trade-off/discrete choice methodology [54]. The single *EQ-5D Index* value can then be used to enable calculation of the health-related quality of life. The EQ-5D-5 L index scores generated using the abovementioned algorithm range from − 0.22 to 1, with the maximum score of 1 indicating the best health quality of life. The EQ-5D Index demonstrated good internal consistency in this study (Cronbach’s α = 0.76).

*Burden.* Caregivers’ level of burden was measured by the 12 item Short Form Zarit Burden Interview (ZBI-12) [55]. The ZBI-12 has been used to evaluate burden in different caregiving contexts with a five-point Likert scale ranging from ‘never’ (0) to ‘always’ (4). Total score is the sum of the items (maximum score = 48). The internal consistency was good (Cronbach’s α =.88).

*Depression.* The Centre for Epidemiological Studies Depression Scale (CESD-10) [56] is a short form questionnaire that consists of 10 items (from the original 20) with response options of 0 to 3 [(0 = Rarely or none of the time (less than 1 day); 1 = Some or a little of the time (1–2 days); 2 = Occasionally or a moderate amount of time (3–4 days); 3 = Most or all of the time (5–7 days)]. The time frame is ‘during the past week’. The total score is calculated by summing the 10 items, with higher scores indicating higher degrees of depressive symptoms (maximum score = 30). The CESD was designed to measure depressive experiences in the general population. In the current study the measure demonstrated good consistency (Cronbach’s α = 0.87).

Measures that were not validated and available in the required language (i.e., RFS, MECS, Willingness to Care Scale, The GAINS Scale) were translated into national languages applying forward and backward translation procedures.

### Data analysis

Responses to the survey were coded and analysed in SPSS (version 27; IBM Corporation, Armonk, NY). Missing data was assumed to be missing at random and constituted less than 6% of the full data [57, 58], therefore hot-deck imputations were performed on all scales [59, 60]. Imputed data was used for all analyses except for demographic variables for which data from complete cases was used. Sensitivity analyses were conducted to investigate whether any effects detected in inferential tests depended on the missing value imputations. There were no significant differences identified.

Assumptions regarding multicollinearity, singularity, linearity, and homoscedasticity were met. With regards to the assumption of normality, various scores on different scales were not normally distributed, however, parametric analyses were performed for all of the scales according to the central limit theorem (i.e., given the sample size, the consequences of violating the assumptions of normality are considered to be trivial [61]). Moreover, in the subsequent analyses, where appropriate, bootstrapping was applied as an alternative to parametric estimates when assumptions of normality were questioned. A few legitimate outliers were detected, i.e., these outliers were not considered to be data entry errors, but actual participant values). Data analyses therefore included these outliers.

Descriptive analyses were conducted (i.e., means and standard deviations for continuous variables, and frequencies and percentages for categorical variables) to describe the sample. One-way ANOVAs and chi-square tests were conducted to examine cross-country differences in demographic composition (e.g., in caregiver’s age, intensity of care, etc.). Finally, ANCOVA tests examined cross-country differences in key psychosocial and outcome variables, controlling for the effect of demographics as appropriate.

## Results

### Sample characteristics

The data from the ENTWINE survey analysed in this article included 879 caregivers from seven countries (Greece, Israel, Italy, the Netherlands, Poland, Sweden, and the UK), with Germany and Ireland excluded from the analysis due to low recruitment numbers (*N* = 25, *N* = 42, respectively). Characteristics of caregiver participants and their care recipients overall and by country are presented in Table 2. Caregivers had a mean age of 56 years and were predominantly women (87%). The youngest mean age was reported in Israel (51 years) and the oldest in Sweden (61 years). Most participants (83%) had at least post-secondary education. Half (50%) of the caregivers were employed, with lower percentages in the UK (34%). The majority (70%) were married or in a partnership. Most caregivers (74%) fell into the lowest income level, with variations across countries and a wide range (49% in Israel to 97% in Greece). Around 59% of participants shared a household with their care recipient(s). Religion was not assessed in Italy and Sweden, while ethnicity was not assessed in Italy, Sweden, and the Netherlands (constituting 31% of the total sample). Among the assessed participants, 65% considered themselves religious. The predominant ethnicity among the remaining countries was British or Irish (38%), followed by Eastern and Central European (21%), Mediterranean (19%), Jewish (16%), and other ethnic groups (6%). Around two-thirds of caregivers did not receive help or support from services, and 85% did not receive welfare benefits. Whilst most caregivers reported being the only caregiver for the care recipient (59%), some did share caring responsibilities (41%). Among those who shared care, 60% identified themselves as the primary caregiver. The majority (84%) considered themselves the primary caregiver, and 42% had provided care to another person in the past. Most caregivers (87%) had provided care for at least a year, with variations across countries. On average, caregivers provided 54.04 h of care per week, with variations across countries. Around 55% of caregivers reported no personal health condition. Caregivers were providing care mostly to parents/parents-in-law (46%) or to partners (32%).

Care recipients were mostly females (51%), with a mean age of 70 years. According to the KATZ Index, care recipients fell between full and partial dependence for activities of daily living (M = 2.93), with the highest independence in the Netherlands and Sweden (KATZ Index M = 3.56; 3.42, respectively) and the lowest in Italy and Poland (KATZ Index M = 2.25; 2.20, respectively). The most common health condition reported among care recipients was ‘other’ (40%), followed by cardiovascular conditions (36%), cognitive or memory impairment (32%), stroke or cerebral vascular disease (17%), diabetes (17%), cancer (16%), and rheumatic health conditions (13%). The remaining conditions were reported in less than 10% of care recipients. Around 40% of caregivers provided care for individuals with a single health condition; around 57% of caregivers provided care for individuals with multiple chronic health conditions, while 3% reported providing care due to the care recipient’s old age, frailty or following an injury.

**Table 2:** Cross-country and overall demographic data for informal caregivers and their care recipients

		Greece		Israel		Italy		Netherlands		Poland		Sweden		UK		N	% or M(SD)
		N	% or M(SD)	N	% or M(SD)	N	% or M(SD)	N	% or M(SD)	N	% or M(SD)	N	% or M(SD)	N	% or M(SD)		
**Informal caregivers’ characteristics**																	
**Age**		80	53.58 (11.48)	125	50.85 (15.97)	187	53.13 (10.91)	189	57.69 (10.42)	69	52.33 (11.95)	90	60.97 (12.65)	139	60.02 (11.49)	879	55.66 (12.53)
**Gender**	**Female**	68	85%	101	81%	161	87%	171	90%	62	90%	76	84%	122	88%	761	87%
	**Male**	12	15%	24	19%	24	13%	17	9%	7	10%	14	16%	16	12%	114	13%
	**Other**	0	0%	0	0%	1	1%	1	1%	0	0%	0	0%	1	1%	3	0%
**Education**	**Primary**	2	3%	0	0%	0	0%	2	1%	0	0%	3	3%	1	1%	8	1%
	**Secondary**	19	24%	22	18%	15	8%	28	15%	8	12%	14	16%	14	10%	120	14%
	**Post-secondary vocational education**	15	19%	16	13%	79	42%	134	71%	12	17%	23	26%	43	31%	322	36%
	**Post-secondary academic education**	43	54%	87	70%	93	50%	23	12%	41	59%	47	52%	80	58%	414	47%
	**Not listed or other**	1	1%	0	0%	0	0%	2	1%	8	12%	3	3%	1	1%	15	2%
**Employed**	**No**	31	39%	46	37%	93	50%	97	51%	30	44%	48	53%	92	66%	437	50%
	**Yes**	49	61%	79	63%	94	50%	92	49%	39	56%	42	47%	47	34%	442	50%
**Other informal caregiver(s)**	**No**	38	48%	51	41%	105	56%	130	69%	36	52%	66	73%	93	67%	519	59%
	**Yes**	42	53%	74	59%	82	44%	59	31%	33	48%	24	27%	46	33%	360	41%
**Provided care to other CR in the past**	**No**	46	58%	80	64%	125	67%	74	39%	45	65%	63	70%	75	54%	508	58%
	**Yes**	34	43%	45	36%	62	33%	115	61%	24	35%	27	30%	64	46%	371	42%
**Help and support from services**	**No**	51	64%	59	48%	111	59%	91	50%	51	74%	54	60%	100	73%	517	60%
	**Yes**	29	36%	63	52%	76	41%	92	50%	18	26%	36	40%	37	27%	351	40%
**Relationship of CG to CR**	**spouse/partner**	7	9%	19	15%	44	24%	84	44%	11	16%	55	61%	57	41%	277	32%
	**parent/parent-in-law**	59	74%	70	56%	106	57%	66	35%	42	61%	17	19%	52	37%	412	46%
	**daughter/son**	6	8%	5	4%	24	13%	17	9%	2	3%	13	14%	15	11%	82	10%
	**another family member**	6	7.5%	23	18.4%	9	4.8%	9	4.8%	9	13.0%	3	3.3%	7	5.0%	66	7.5%
	**non-relative member**	2	3%	8	6%	4	2%	13	7%	5	7%	2	2%	8	6%	42	5%
**Sharing the same household with CR**	**No**	37	46%	82	66%	62	33%	87	46%	29	42%	26	29%	42	30%	365	41%
	**Yes**	43	54%	43	34%	125	67%	102	54%	40	58%	64	71%	97	70%	514	59%
**Choice in taking on the responsibility of caring**	**No**	61	76%	82	66%	134	72%	158	84%	46	67%	78	87%	103	74%	662	75%
	**Yes**	19	24%	43	34%	53	28%	31	16%	23	33%	12	13%	36	26%	217	25%
**Total period of caregiving in weeks** **(range in years)**		80	315.53 (0.01–49)	125	243.49 (0.01–30)	187	406.82 (0.8–50)	189	397.00 (0.07–30)	69	298.37 (0.17–43)	90	310.27 (0.17–31)	139	446.83 (0.05–41)	879	361.10 (0.01–50)
**Total number of hours spent on caregiving per last week**		80	49.77 (44.78)	125	30.93 (39.14)	187	73.71 (70.41)	189	40.70 (41.33)	69	72.50 (56.10)	90	49.05 (44.49)	139	63.01 (48.03)	879	54.04 (53.43)
**CG’s health condition**	**A physical impairment or disability**	6	8%	20	16%	11	6%	30	16%	2	3%	13	14%	20	14%	102	12%
	**Sight or hearing loss**	1	1%	13	10%	7	4%	15	8%	1	1%	13	14%	12	9%	62	7%
	**A mental health problem or illness**	7	9%	14	11%	11	6%	18	10%	2	3%	7	8%	18	13%	77	9%
	**A learning disability or difficulty**	2	3%	5	4%	3	2%	4	2%	2	3%	5	6%	1	1%	22	2%
	**A long-standing illness**	12	15%	10	8%	22	12%	30	16%	18	26%	15	17%	26	19%	133	16%
	**Multimorbidity**	2	3%	9	7%	23	12%	15	8%	7	10%	8	9%	6	4%	70	8%
	**Other condition or disability**	7	9%	11	9%	22	12%	28	15%	2	3%	14	16%	19	14%	103	12%
	**No conditions or disabilities**	53	66%	64	51%	115	62%	91	48%	44	64%	45	50%	72	52%	484	55%
**Care recipients’ characteristics**																	
**Age of CR**		80	76.27 (18.72)	125	73.97 (19.60)	187	68.51 (20.66)	189	65.99 (19.27)	69	74.69 (14.48)	90	64.72 (18.59)	139	70.00 (19.43)	879	69.78 (19.51)
**KATZ (ADL) score**		80	2.83 (2.30)	125	3.38 (2.40)	187	2.25 (2.17)	189	3.56 (2.08)	69	2.20 (2.20)	90	3.42 (2.27)	139	2.81 (2.13)	879	2.93 (2.28)
**Gender**	**Female**	62	78%	75	61%	107	57%	74	40%	43	63%	29	32%	56	40%	446	51%
	**Male**	18	23%	49	40%	80	43%	113	60%	25	37%	61	68%	83	60%	429	49%
**Number of CR’s conditions**	**No physical/mental condition**	3	4%	11	9%	7	4%	5	3%	1	1%	2	2%	4	3%	33	3%
	**Single health condition**	38	48%	45	36%	98	52%	69	37%	22	32%	35	39%	42	30%	349	40%
	**Multimorbidity**	39	49%	69	55%	82	44%	115	61%	46	67%	53	59%	93	67%	497	57%

Abbreviations: CG– informal caregivers; CR– care recipients

### Cross-country differences in study variables

Table 3 presents cross-country differences in the psychosocial variables. The description presented below is based on post hoc analyses using the Bonferroni test for multiple comparisons following the conducted ANCOVAs.^3^

### Extrinsic motivations

Extrinsic motivations were typically at a high level (maximum possible score = 35) although no statistically significant differences between the countries were detected in extrinsic motivations (*p* =.088).

### Intrinsic motivations

Intrinsic motivations were lower in all countries than extrinsic motivations but were consistently at a moderate-high level (maximum possible score = 30). Caregivers in Sweden scored lower on intrinsic motivations than caregivers in the UK (*p* =.041, 95% C.I.=-3.59, − 0.03).

### Willingness to provide nursing care

Willingness to provide nursing care was at a moderate-high level (maximum possible score = 5), lowest in Sweden, highest in Italy and with significant variation between countries. Caregivers in Sweden scored lower on willingness to provide nursing care than caregivers in: Greece (*p* =.001, 95% C.I.=-1.45, − 0.57), Israel (*p* =.001, 95% C.I.=-1.06, − 0.26), Italy (*p* =.001, 95% C.I.=-1.42, − 0.68), the Netherlands (*p* =.001, 95% C.I.=-1.28, − 0.55), Poland (*p* =.002, 95% C.I.=-1.02, − 0.12) and the UK (*p* =.001, 95% C.I.=-1.35, − 0.60). Caregivers in Italy scored higher on willingness to provide nursing care than caregivers in Israel (*p* =.009, 95% C.I.=0.05, 0.72), Poland (*p* =.003, 95% C.I.=0.09, 0.87). Also, caregivers in Poland scored lower on willingness to provide nursing care than caregivers in the UK (*p* =.046, 95% C.I.=-0.82, − 0.01).

### Willingness to provide emotional care

Willingness to provide emotional care was moderate to high (maximum possible score = 5), the lowest in Poland, and the highest in the UK. Caregivers in Poland differed significantly to caregivers in Israel (*p* =.001, 95% C.I.=-0.87, − 0.06), the Netherlands (*p* =.001, 95% C.I.=-0.84, − 0.17) and the UK (*p* =.001, 95% C.I.=-0.83, − 0.17). The higher willingness to provide emotional care in the UK differed significantly to caregivers in Sweden (*p* =.029, 95% C.I.=0.01, 0.62). Similarly, caregivers in the Netherlands scored higher on willingness to provide emotional care than caregivers in Sweden (*p* =.019, 95% C.I.=0.02, 0.61).

### Willingness to provide instrumental care

Willingness to provide instrumental care was moderate to high (maximum possible score = 5), with the lowest in Sweden, and the highest in the UK. Caregivers in Sweden scored lower on willingness to provide instrumental care than those in all other countries, i.e.: Greece (*p* =.001, 95% C.I.=-1.26, − 0.61), Israel (*p* =.001, 95% C.I.=-1.17, − 0.58), Italy (*p* =.001, 95% C.I.=-1.18, − 0.63), the Netherlands (*p* =.001, 95% C.I.=-1.02, − 0.47), Poland (*p* =.001, 95% C.I.=-0.98, − 0.31) and the UK (*p* =.001, 95% C.I.=-1.21, − 0.65).

### Global willingness to provide care

Levels of overall willingness to provide care were moderate to high (maximum possible score = 5); again lowest in Sweden and highest in the UK. Caregivers in Sweden scored lower on global willingness than caregivers in Greece (*p* =.001, 95% C.I.=-0.97, − 0.33), Israel (*p* =.001, 95% C.I.=-0.88, − 0.30), Italy (*p* =.001, 95% C.I.=-0.95, − 0.41), the Netherlands (*p* =.001, 95% C.I.=-0.94, − 0.41), Poland (*p* =.020, 95% C.I.=-0.68, − 0.02) and the UK (*p* =.001, 95% C.I.=-1.00, − 0.45). Caregivers in Poland scored lower on global willingness than caregivers in Italy (*p* =.008, 95% C.I.=-0.61, − 0.04), the Netherlands (*p* =.022, 95% C.I.=-0.62, − 0.02) and the UK (*p* =.002, 95% C.I.=-0.67, − 0.07).

**Table 3:** Descriptive data for study variables and ANCOVA analyses

	Country of residence							Total		ANCOVA analyses	
	Greece	Israel	Italy	Netherlands	Poland	Sweden	UK	N	M(SD)	Country, F	*p* value
**Extrinsic Motivations to Care**	28.33 (4.26)	26.66 (5.23)	27.99 (4.97)	26.60 (5.31)	27.53 (4.93)	28.74 (4.67)	28.06 (4.68)	879	27.47 (5.04)	2.97	*p* =.088
**Intrinsic Motivations to Care**	25.41 (3.88)	24.50 (3.92)	24.97 (4.21)	25.06 (4.70)	23.62 (5.15)	23.85 (3.89)	25.54 (3.59)	879	24.78 (4.28)	3.84	*p* <.007**
**Willingness - nursing care**	4.32 (0.85)	3.80 (1.02)	4.44 (0.71)	4.23 (0.96)	3.93 (0.84)	3.39 (0.83)	4.35 (0.74)	879	4.13 (0.92)	15.22	*p* <.001***
**Willingness - emotional care**	4.29 (0.79)	4.42 (0.61)	4.38 (0.63)	4.55 (0.68)	4.04 (0.77)	4.29 (0.77)	4.57 (0.59)	879	4.40 (0.69)	6.63	*p* <.001***
**Willingness - instrumental care**	4.50 (0.62)	4.34 (0.69)	4.54 (0.58)	4.36 (0.77)	4.23 (0.71)	3.74 (0.65)	4.60 (0.52)	879	4.38 (0.70)	20.52	*p* <.001***
**Willingness - global score**	4.35 (0.67)	4.19 (0.67)	4.44 (0.52)	4.39 (0.71)	4.09 (0.65)	3.80 (0.61)	4.50 (0.48)	879	4.29 (0.67)	15.04	*p* <.001***
**Familial interconnectedness (RFS)**	27.60 (6.42)	32.57 (6.29)	29.27 (6.92)	27.82 (6.16)	29.33 (5.87)	25.21 (6.72)	29.17 (5.21)	879	28.77 (6.58)	9.85	*p* <.001***
**Extended family support (RFS)**	7.08 (3.02)	8.14 (2.54)	6.71 (3.01)	5.92 (2.77)	7.73 (2.95)	4.82 (3.15)	7.15 (2.44)	879	6.77 (2.96)	10.65	*p* <.001***
**Familial obligations (RFS)**	7.63 (2.98)	8.23 (3.02)	7.63 (3.19)	6.31 (2.71)	7.37 (3.39)	5.64 2.87)	7.30 (2.70)	879	7.23 (3.08)	7.75	*p* <.001***
**Familism (RFS total score)**	42.47 (11.11)	48.80 (10.21)	43.51 (11.32)	39.58 (10.10)	44.78 (10.35)	35.70 (11.22)	43.92 (8.52)	879	42.74 (10.95)	11.52	*p* <.001***
**Self-transcendence (PVQ-21)**	1.98 (0.64)	2.2 (0.78)	2.11 (0.73)	2.24 (0.60)	2.12 (0.63)	2.31 (0.72)	2.20 (0.79)	879	2.18 (0.72)	2.72	*p* =.390
**Self-enhancement (PVQ-21)**	4.05 (1.12)	3.42 (1.00)	3.65 (1.04)	4.11 (0.98)	4.24 (0.85)	4.43 (0.86)	4.32 (0.89)	879	4.00 (1.03)	8.79	*p* =.001***
**Presence of meaning (MLQ)**	24.75 (5.16)	26.30 (5.59)	23.50 (6.68)	24.12 (5.20)	23.65 (7.00)	23.64 (6.71)	22.77 (6.64)	879	23.94 (6.24)	2.87	*p* =.004**
**Search for meaning (MLQ)**	23.28 (5.86)	22.36 (7.58)	19.55 (7.56)	20.11 (6.17)	25.44 (5.80)	18.15 (7.44)	17.64 (6.84)	879	20.28 (7.32)	9.30	*p* =.001***
**Illness threat (IPQ score)**	52.62 (8.55)	52.87 (8.33)	56.17 (9.48)	56.73 (7.80)	57.79 (9.82)	57.81 (8.17)	59.07 (8.75)	879	56.30 (8.90)	5.58	*p* <.001***
**Wellbeing (WHO-5 score)**	44.80 (23.80)	60.73 (21.93)	40.36 (22.71)	52.35 (23.56)	30.72 (22.19)	43.86 (23.86)	42.82 (23.40)	879	46.01 (24.33)	8.58	*p* <.001***
**Caregiver gains (GAINS score)**	10.63 (6.66)	10.32 (6.60)	10.81 (6.37)	14.43 (6.25)	11.60 (6.13)	15.32 (6.21)	16.51 (6.57)	879	13.06 (6.77)	15.01	*p* <.001***
**Health quality of life (EQ-5D-5 L utility index)**	0.81 (0.16)	0.89 (0.11)	0.71 (0.25)	0.78 (0.19)	0.84 (0.16)	0.83 (0.12)	0.80 (0.16)	879	0.79 (0.19)	6.07	*p* <.001***
**Burden (ZBI score)**	22.25 (9.53)	16.68 (8.52)	20.77 (8.88)	18.43 (8.97)	22.86 (9.12)	23.90 (9.52)	22.51 (9.17)	879	20.66 (9.33)	7.04	*p* <.001***
**Depression (CES-D score)**	12.13 (6.35)	8.81 (5.63)	12.77 (6.62)	9.78 (5.62)	13.91 (7.51)	12.52 (6.17)	12.61 (6.76)	879	11.64 (6.49)	5.07	*p* <.001***

ANCOVA tests controlled for the effects of age, employment, experience providing care, having support from other caregivers, caregiver educational levels, caregiver-care recipient relationship type, caring period, intensity of care, KATZ Index, care recipient’s age and gender, and the number of care recipient’s health conditions

**p <.01; ***p <.001

### Familism

Familism varied across countries from a moderate level in Sweden to a higher, but still moderate, level in Israel (maximum available score = 84). Caregivers in all countries included in analysis^4^ apart from the Netherlands scored higher on familism than caregivers in Sweden (GR, *p* =.023, 95% C.I.=0.37, 11.21; IL, *p* =.001, 95% C.I.=8.24, 18.11; IT, *p* =.001, 95% C.I.=2.94, 12.09; PL, *p* =.001, 95% C.I.=2.64, 13.78; UK, *p* =.001, 95% C.I.=3.60, 12.83). Caregivers in Israel scored higher on familism than caregivers in Italy (*p* =.001, 95% C.I.=1.48, 9.83), the Netherlands (*p* =.001, 95% C.I.=5.33, 14.08), Greece (*p* =.001, 95% C.I.=2.45, 12.31) and the UK (*p* =.012, 95% C.I.=0.55, 9.37). Caregivers in Italy scored higher on familism than caregivers in the Netherlands (*p* =.025, 95% C.I.=0.23, 7.85). Caregivers in the Netherlands scored lower on familism than caregivers in the UK (*p* =.005, 95% C.I.=-8.72, − 0.765).

### Familial interconnectedness

Familial interconnectedness levels were generally moderate (maximum possible score = 48), with the lowest level in Sweden and the highest in Israel, both still within moderate ranges. Caregivers in all countries scored lower on familial interconnectedness than caregivers in Israel (GR, *p* =.001, 95% C.I.=-8.50, -2.45; IT, *p* =.001, 95% C.I.=-6.10, − 0.98; NL, *p* =.001, 95% C.I.=-7.89, -2.53; PL, *p* =.009, 95% C.I.=-6.77, − 0.46; SE, *p* =.001, 95% C.I.=-10.46, -4.40; UK, *p* =.003, 95% C.I.=-6.06, − 0.66). Also, caregivers in Italy, Poland and the UK scored higher on familial interconnectedness than caregivers in Sweden (IT, *p* =.001, 95% C.I.=1.08, 6.69; PL, *p* =.013, 95% C.I.=0.40, 7.23; UK, *p* =.001, 95% C.I.=1.24, 6.90).

### Family support

Levels of perceived family support ranged from the boundary between low and moderate, and moderate (maximum possible score = 16), with the lowest level seen in Sweden and the highest in Israel. Caregivers in all countries except the Netherlands (Greece, Israel, Italy, Poland and the UK) scored higher on family support than caregivers in Sweden (GR, *p* =.001, 95% C.I.=0.49, 3.40; IL, *p* =.001, 95% C.I.=1.83, 4.48; IT, *p* =.001, 95% C.I.=0.38, 2.83; PL, *p* =.001, 95% C.I.=1.06, 4.05; UK, *p* =.001, 95% C.I.=1.03, 3.50). Also, caregivers in Israel, Poland, and the UK scored higher on family support than caregivers in the Netherlands (IL, *p* =.001, 95% C.I.=1.08, 3.43; PL, *p* =.004, 95% C.I.=0.29, 3.01; UK, *p* =.002, 95% C.I.=0.29, 2.43). Caregivers in Israel scored higher on family support than caregivers in Italy (*p* =.001, 95% C.I.=0.43, 2.67).

### Familial obligations

Levels of familism were moderate in all countries (maximum score = 20), with between-country differences seen. Caregivers in Sweden scored significantly lower on familial obligation than caregivers in Greece, Israel, Italy, and the UK (*p* =.003, 95% C.I.=0.34, 3.43; *p* =.001, 95% C.I.=1.32, 4.13; *p* =.001, 95% C.I.=0.79, 3.39; *p* =.002, 95% C.I.=0.34, 2.97; respectively). Caregivers in Israel and Italy scored higher on familial obligations than caregivers in the Netherlands (*p* =.001, 95% C.I.=0.79, 3.29; *p* =.001, 95% C.I.=0.31, 2.49; respectively).

### Self-transcendence values

Levels of self-transcendence values were typically low (< 3 in a maximum score of 6), although highest in Sweden, and lowest in Greece. No statistically significant differences between the countries were detected in self-transcendence values (*p* =.390).

### Self-enhancement values

Self-enhancement values were higher (> 3 in a maximum possible score of 6) than levels of self-transcendence values, with several between-country differences. Caregivers in Israel scored lower on self-enhancement values than caregivers in the Netherlands (*p* =.021, 95% C.I.=-0.85, − 0.03), Poland (*p* =.001, 95% C.I.=-1.18, − 0.21), Sweden (*p* =.001, 95% C.I.=-1.27, − 0.34) and the UK (*p* =.001, 95% C.I.=-1.09, − 0.26). Similarly, caregivers in Italy scored lower on self-enhancement values than caregivers in the Netherlands (*p* =.037, 95% C.I.=-0.72, − 0.01), Poland (*p* =.001, 95% C.I.=-1.07, − 0.17), Sweden (*p* =.001, 95% C.I.=-1.16, − 0.30) and the UK (*p* =.001, 95% C.I.=-0.97, − 0.23).

### Presence of meaning

Caregivers reported moderate to high presence of meaning (maximum possible score = 35) with limited between-country variation apart from caregivers in Israel scoring higher on the presence of meaning than caregivers in the UK (*p* =.001, 95% C.I.=0.84, 6.10).

### Search for meaning

Levels of searching for meaning were typically lower (maximum possible score = 35) than levels of the presence of meaning, with several between country differences. Caregivers in Poland scored higher on the search for meaning than caregivers in Italy (*p* =.001, 95% C.I.=2.48, 9.00), the Netherlands (*p* =.001, 95% C.I.=1.14, 8.05), Sweden (*p* =.001, 95% C.I.=2.55, 10.13) and the UK (*p* =.001, 95% C.I.=3.95, 10.82). Caregivers in the UK scored lower on the search for meaning than caregivers in Greece (*p* =.001, 95% C.I.=-8.74, -2.07), Israel (*p* =.001, 95% C.I.=-7.24, -1.24) and the Netherlands (*p* =.036, 95% C.I.=-5.49, − 0.07). Also, caregivers in Italy and Sweden scored lower on the search for meaning than caregivers in Greece (*p* =.004, 95% C.I.=-6.89, − 0.63; *p* =.006, 95% C.I.=-8.05, − 0.67; respectively).

### Illness threat

Levels of perceived illness threat were typically moderate (maximum possible score = 80), although highest in the UK, and lowest in Greece and Israel. Caregivers in the UK scored significantly higher on perceived illness threat than caregivers in Greece (*p* =.001, 95% C.I.=2.23, 10.02), and Israel (*p* =.002, 95% C.I.=0.96, 7.97) and Italy (*p* =.001, 95% C.I.=0.93, 7.13). Caregivers in Sweden and Poland scored higher on perceived illness threat than caregivers in Greece (*p* =.005, 95% C.I.=0.82, 9.44; *p* =.044, 95% C.I.=0.05, 8.87). Caregivers in the Netherlands also scored higher on perceived illness threat than caregivers in Greece (*p* =.001, 95% C.I.=1.33, 8.99), Israel (*p* =.045, 95% C.I.=0.03, 6.99) and Italy (*p* =.042, 95% C.I.=0.04, 6.10).

### Wellbeing

Wellbeing varied highly between countries, with the lowest reported in Poland and the highest in Israel. Wellbeing was low (i.e., below the cut-off score of ≤ 50) in Poland, Greece, Italy, Sweden, the UK, and normative (i.e., above the cut-off score of > 50) for Israel and the Netherlands. Caregivers in Israel reported higher wellbeing than caregivers in Greece (*p* =.001, 95% C.I.=3.65, 25.20), Italy (*p* =.001, 95% C.I.=5.46, 23.71), Poland (*p* =.001, 95% C.I.=13.43, 35.95), Sweden (*p* =.001, 95% C.I.=6.37, 27.94) and the UK (*p* =.001, 95% C.I.=6.45, 25.71). Caregivers in Poland scored lower on wellbeing than caregivers in the Netherlands (*p* =.001, 95% C.I.=-27.26, -5.11).

### Gains

Reported gains of caregiving were low to moderate (maximum possible score = 30), ranging from 10.3 in Israel to 16.5 in the UK. Caregivers in the UK scored significantly higher on gains than caregivers in Greece (*p* =.001, 95% C.I.=3.09, 9.06), Israel (*p* =.001, 95% C.I.=3.84, 9.23), Italy (*p* =.001, 95% C.I.=3.31, 8.07) and Poland (*p* =.001, 95% C.I.=1.94, 8.11). Caregivers in the Netherlands reported significantly more gains than caregivers in Greece (*p* =.001, 95% C.I.=1.81, 7.70), Israel (*p* =.001, 95% C.I.=2.54, 7.89), Italy (*p* =.001, 95% C.I.=2.04, 6.70) and Poland (*p* =.005, 95% C.I.=0.61, 6.80). Similarly, caregivers in Sweden scored higher on gains than caregivers in Greece (*p* =.001, 95% C.I.=1.70, 8.40), Israel (*p* =.001, 95% C.I.=2.54, 8.57), Italy (*p* =.001, 95% C.I.=1.91, 7.50) and Poland (*p* =.005, 95% C.I.=0.64, 7.45).

### Health-related quality of life

Health-related quality of life was moderate to high (maximum possible score = 1), with Israel highest and Italy lowest. Caregivers in Israel scored higher on health quality of life than caregivers in Italy (*p* =.001, 95% C.I.=0.06, 0.21) and the Netherlands (*p* =.002, 95% C.I.=0.02, 0.18). Caregivers in Italy scored lower on the health quality of life than caregivers in Poland (*p* =.001, 95% C.I.=-0.20, − 0.03), Sweden (*p* =.010, 95% C.I.=-0.17, − 0.01) and the UK (*p* =.014, 95% C.I.=-0.15, − 0.01).

### Burden

Levels of reported burden were low to moderate (maximum possible score = 48). The lowest levels in Israel differed significantly from the highest in Sweden (*p* =.001, 95% C.I.=4.11, 12.56), and Sweden also differed from Italy (*p* =.001, 95% C.I.=2.19, 10.03) and the Netherlands (*p* =.001, 95% C.I.=1.66, 9.41). Caregivers in Israel scored lower on burden than caregivers in Greece (*p* =.001, 95% C.I.=-9.64, -1.20) and the UK (*p* =.001, 95% C.I.=-9.28, -1.73).

### Depression

Levels of depression were low to moderate (maximum possible score = 30), the lowest in Israel and the highest in Poland. Caregivers in Israel scored lower on depression than caregivers in Greece (*p* =.023, 95% C.I.=-6.04, − 0.20), Italy (*p* =.006, 95% C.I.=-5.39, − 0.44), Poland (*p* =.002, 95% C.I.=-6.90, − 0.79), Sweden (*p* =.001, 95% C.I.=-7.19, -1.34) and the UK (*p* =.001, 95% C.I.=-6.37, -1.14). Caregivers in Sweden scored higher on depression than caregivers in the Netherlands (*p* =.026, 95% C.I.=0.15, 5.52).

## Discussion

This paper reports cross-country variations in caregiver experiences, focusing on rarely described caregiver motivations, willingness to care, cultural and personal values, meaning in life, perceived illness threat and caregiver experiences of wellbeing, gain, health-related quality of life, burden and depression. The study adopted an exploratory approach. Findings pertaining to variations across 6 European countries and Israel (included in ANCOVAs which controlled for several covariates, see Table 3 and footnote) are presented^5^.

Cross-country variations in caregiver *motivations* and *willingness to care* have not been examined previously. The findings show that extrinsic and intrinsic motivations were at moderate-high levels in all countries with only limited between-country variation. The measure of motivations to provide care (see Methods section) was created in the UK [16], and was translated for the first time to other languages for the current study. Therefore, to draw comparisons, we can only refer to UK-based studies applying this scale [16, 62, 63] and in each of these caregivers also scored on average moderate-high on extrinsic and intrinsic motivations for caring. These previous findings as well as our current findings are congruent with the proposal that extrinsic and intrinsic motivations are not mutually exclusive [15], i.e., a high level of extrinsic motivations does not imply an absence nor a low level of intrinsic motivations and vice versa. Extrinsic and intrinsic motivations should be considered on two dimensions, and can co-exist.

Willingness to care (globally) and its components (nursing, emotional and instrumental willingness) were at moderate to high mean levels with cross-country variations observed. A general pattern found lower global willingness to care in caregivers in Sweden, the highest global willingness to care amongst caregivers in the UK, Italy, and the lowest levels of emotional willingness amongst caregivers in Poland. This difference between Poland and the rest of the countries in terms of emotional willingness could be considered in the context of Poland lacking a national policy to underpin caregiver support [39, 40]. However, caregivers in Sweden, a country with potentially the most comprehensive and developed caregiver support system in our sample, reported significantly lower, albeit still moderate, mean levels of global willingness to care as compared with other countries. The dominant trend of policy reforms has been centred around an increasing shift of the responsibility of care from the state to caregivers [2, 64], including Sweden [65, 66]. Although noted for its well-developed caregiver support, the shift of responsibility of care to families coupled with cutbacks on social spending in Sweden [66] may potentially explain their lower willingness to offer informal care as opposed to caregivers in other countries.

Between country differences in mean levels of *caregiver values* (familism and personal values) could potentially be interpreted in the context of both cultural and policy differences existing between southern countries, for example Israel and Greece, and northern countries, such as Sweden or the Netherlands [67, 68]. Caregivers in Sweden and the Netherlands reported lower mean levels of familism across all subscales (familial interconnectedness, family support, familial obligations) than caregivers in Israel. The highest level of familism (and subcomponents) reported in Israel may be due to the major significance ascribed to older people in this country’s religious tradition as well as to the presence of particular sources of stress such as for example recurrent wars that may play a role in strengthening family relations, family values and obligations [69].

The mean levels of self-transcendence values were rather low across all countries; however, it is difficult to compare this finding with that of other studies as they do not exist. Self-enhancement values were typically higher across the national samples than self-transcendence values; the highest in Sweden, a country typically characterised by individualism, and much lower in Israel, a country characterised by collectivism [43]. The differences here between northern and southern countries may reflect differences in health and social care systems underpinning caregiver support [67, 68], yet in terms of the most notable difference between Sweden and Israel, where both countries are considered to provide high levels of statutory social care, we would tend to ascribe the difference more to cultural context rather than the availability of social care.

There was limited between-country variation in *the presence of meaning*, with all countries reporting moderate to high levels of the presence of meaning. However, there was cross-country variation in *searching for meaning* in life with caregivers from the poorer countries of Poland and Greece reporting the highest mean levels of the search for meaning in life, and the relatively wealthier UK reporting the lowest levels 6. This replicates general population patterns seen in the 132 countries represented in Gallup World Poll data [24] although other cross-country comparisons of meaning in life have been limited [70], e.g., by the inclusion of a small number of countries [71], or the use of dichotomous single-items to assess meaning [24]. The findings reported here are from seven countries using a validated meaning in life measure, are specific to caregivers, and thus add usefully to caregiving literature.

Studies of caregiver *illness beliefs* (in terms of perceived threat) are limited and have not to date considered cross-country variations. In this study, the highest levels of perceived illness threat were noted in the UK and Sweden, countries scoring high on individualism characteristics [44]. In contrast, the lowest levels of perceived illness threat were reported in Israel and Greece, the most collectivist countries in our sample [44]. Israeli caregivers also scored highest on familism and filial obligations which are particularly dominant values in collectivist cultures [72]. Previous studies have shown that caregivers from collectivist cultures tend to ground their illness beliefs in familial values, e.g., they ascribe the potential illness threat more to family stress, worry, pressure, wrongdoing, and family discord rather than to the physical/mental damage caused by illness [72, 73].^7^ However, the extent to which differences in illness perceptions can truly be ascribed to culture is not clear given the range of other potential influences presented here and elsewhere [e.g., 69].

Caregiver experiences of *wellbeing*, *gain*, *health-related quality of life*, *burden* and *depression* have rarely been studied from a cross-national perspective [10, 68]. To begin with, the low level of caregiver *wellbeing* found across most of the countries in our study is consistent with previous studies which have reported that caregivers have low levels of wellbeing and lower than non-caregivers [2, 75]. Caregivers from Poland reported the lowest mean levels of wellbeing, and those from Israel reported the highest mean levels. Caregivers in Israel also reported the highest mean levels of health-related quality of life. Although both countries (Poland and Israel) are family-based countries [10, 12], the national caregiver support policies may be influential here, with Israel’s higher commitment to supporting caregivers translating to better wellbeing than in Poland, where caregiver support is underdeveloped [39, 40, 76].^8^

On average, north-western countries reported higher mean levels of *gains* than south-eastern countries. This finding appears congruent with the pattern of differences in health/social care systems underpinning caregiver support between north-west and south-east of Europe [67, 68]. That is, higher provision of caregiver support may be reflected in higher levels of reported gains [10]. Interestingly, however, Israeli caregivers comprise an exception having reported lowest mean levels of gains. It may be that a culturally prescribed value of caregiving in Israel condition these caregivers to see their caregiving as obligatory rather than beneficial and meaningful to them [76]. Israeli caregivers may perceive their duty to care as an important value, and do not expect to ‘gain’ from their role. For example, the most frequent reasons for caregiving in Israel were wanting to help and commitment to care [77].

Cross-country variations were seen in mean levels of *burden* and *depression* with caregivers from Sweden and Poland reporting higher mean levels of burden and depression, and caregivers from Israel reporting lower mean levels. As far as higher mean levels of burden and depression in Poland can be ascribed to limited support for caregivers in this country [39, 40], high mean levels of burden and depression in Sweden were unexpected and there are no existing comparator studies with a Swedish sample to aid interpretation of this finding. Caregivers in Sweden have reportedly been negatively impacted by significant cutbacks in social care services in the last two decades [40, 66], where reductions in nursing and residential care and cutbacks in other social care services are shown to have had negative repercussions for caregivers [65, 66]. This may explain why caregivers in Sweden report more negative consequences of their role in the current findings. A cross-national study comparing adult caregivers in Nordic countries (e.g., Sweden) and Continental/Southern countries showed that the latter group reported greater levels of self-realisation and satisfaction [68]. However, it should be mentioned that mean levels of caregiver burden and depression were still relatively low to moderate in the current study in comparison to other published data [78, 79], and congruent with a more recent multinational study on informal care [5].

### Implications of findings

The findings suggest that potential psychological and social care interventions targeting various caregiver variables (e.g., caregiver wellbeing) may need to consider countrycontext, as countries may differ or be similar with regards to levels of such variables. Different countries may require different or similar approaches. For example, high levels of perceived threat in Sweden and the UK, low levels of emotional willingness to care in Poland warrant more attention for within-country future studies and potential interventions. Future cross-country research is needed to establish what works best for which country or countries.

Appropriate support measures should be accessible to all caregivers to promote their wellbeing and alleviate burden and depression. Our data indicates that countries with underdeveloped caregiver support systems (e.g., Poland) tend to have higher negative caregiver experiences compared to countries with more developed support systems (e.g., the Netherlands). This highlights the importance of addressing caregiver support in European policy agendas. National differences in social protection systems, the amount of benefits, and differences in gender perceptions contribute to variations in caregiver experiences (e.g., of burden or wellbeing) [40]. The findings further suggest that the effectiveness of caregiver support, particularly in enhancing positive caregiver wellbeing, gains and health-related quality of life, may depend on the pattern of family values in society (cultural and personal values).

In terms of future implications for research, further cross-country comparative studies are required to more fully examine the potential relation between polices underpinning caregiver support and caregiver motivations, willingness, cultural and personal values, meaning processes, perceived illness threat, and the variety of other experiences (e.g., wellbeing, burden). These findings further highlight the importance of collecting cross-country samples to better understand their experience. A separate paper will present results pertaining to the relationships between these variables.

### Strengths and limitations

The strength of the study lies in the large amount of descriptive data collected from a diverse sample of caregivers using validated measures across seven countries with differing care systems and differing cultural attitudes around informal care. This diversity likely enhances the generalisability of the results. The response rate achieved in the survey was good, i.e., greater than 40%, which is higher than the average response rate for web-based cohort surveys [80]. In presenting cross-country similarities and differences. ANCOVA tests controlled for many possible confounders, increasing our confidence in the between-country differences that emerged.

Several limitations should be acknowledged. Firstly, the cross-sectional nature of the data restricts causal interpretations, although it allows for detailed cross-country analysis of important psychosocial variables. Secondly, between-country variations may be influenced by sampling differences, including variations in sample size and characteristics such as age, employment status, care experience, time spent caregiving, and intensity of care. Statistical tests controlled for the effect of these (and other) potential confounders to increase the likelihood that any differences could be attributed to the caregiver’s country of residence. However, due to an inadequate level of available research support in Germany and Ireland (that resulted in low *N* = 25 and *N* = 42, respectively), these countries were excluded from the ANCOVAs– decreasing the number of compared countries from nine to seven. Thirdly, the small sample sizes in each country limited the possibility of detailed country-specific analyses. Fourth, considering the dearth of cross-country studies in informal care, particularly in relation to the variables examined in the current study, the scope for comparisons with other cross-country studies was correspondingly limited. The study adopted an exploratory approach. Fifth, it is crucial to recognise that participants with certain demographics, particularly informal caregivers with limited access to the Internet and lower digital literacy, may have been excluded from participation in the web-based survey such as the ENTWINE-iCohort, potentially introducing bias into the study findings [81]. Addressing this limitation remains a challenge in research involving web-based methodologies [82]. Finally, generalisability of the findings is limited by the predominantly female and highly educated caregiver sample, which may introduce bias into our findings. However, the consistent overrepresentation of women in informal care studies [83, 84] reflects their actual predominant role in providing informal care, as seen in European statistics [85].

## Conclusions

Extrinsic and intrinsic motivations, as well as willingness to provide care, were moderate-high across all countries. No consistent picture supporting the relation between (known) national caregiver support policies/country culture and caregiver motivations/willingness was found. Between-country differences in caregiver values and illness perceptions likely stem from differences in country culture, with individualist cultures showing lower familism, higher self-enhancement values, and greater perceived illness threat compared to collectivist countries. Poorer countries reported higher search for meaning in life than wealthier countries. Countries with underdeveloped caregiver support systems generally exhibited higher negative caregiver experiences (burden, depression) and lower positive experiences (well-being, gains), except for Sweden where recent cutbacks in social care services may have contributed to higher, albeit still moderate, caregiver burden and depression. Cross-country differences can be explained to varying degrees by national policies around care (or their absence) and country cultural contexts. Health and social care policies, caregiver assessment, and support planning should consider the diverse caregiving relationships and populations, as caregiver experiences may vary between countries. The findings underscore the complexity of the caregiving experience and highlight the importance of caregiver psychosocial support. Going forward, these results can help inform the development of policies and measures to support caregivers in Europe and Israel.

## Data Availability

All anonymised data will be openly available upon request to the author Val Morrison after September 2023, or on completion of ENTWINE team’s key outputs, if this is earlier. OpenAIRE and Zenodo will be used as data repositories.

## Footnotes

1 For specific and detailed cross-country differences, please see the full articles to which references are provided. Due to length of these type of findings, only a summary is provided in the introduction to emphasise the issue of eventual cross-country variations in the study variables.

2 Germany and Ireland were excluded from the analyses presented in this article as they did not meet the necessary assumptions.

3 Germany and Ireland were excluded from the ANCOVAs as they did not meet the necessary assumptions.

4 Country codes: GR = Greece; IL = Israel; IT = Italy; NL = Netherlands; PL = Poland; SE = Sweden; UK = United Kingdom.

5 ANCOVA tests controlled for several possible cofounders, including care recipient’s age and gender, the number of care recipient’s health conditions to ensure that the differences in variables examined were due to the caregiver’s country of residence.

6 The level of wealthiness of the country was based on the data from the World Bank with regards to PPP GDP [86], i.e., GDP per capita based on PPP (purchasing power parity). The following PPP GDP per capita have been reported for the two set of countries between which the highest significant differences have been noted: the United Kingdom (3,12); Poland (1,29) and Greece (0,29).

7 As a reminder, ANCOVA tests controlled for several possible cofounders (see Table 3 and Methods).

8 ANCOVA tests controlled for several possible cofounders, including the intensity of care (i.e., the number of hours spent on caregiving per last week).
